# Characterization of Rice Volatile Secondary Metabolites and Their Role in Modulating the Behavior of the Brown Planthopper (*Nilaparvata lugens* Stål)

**DOI:** 10.3390/insects17030253

**Published:** 2026-02-27

**Authors:** Lang Yang, Li-Fei Huang, Wen-Jie Huang, Guy Smagghe, Jian-Jun Jiang, En-Hai Chen

**Affiliations:** 1 Guangxi Key Laboratory of Biology for Crop Diseases and Insect Pests, Key Laboratory of Green Prevention and Control on Fruits and Vegetables in South China Ministry of Agriculture and Rural Affairs, Plant Protection Research Institution, Guangxi Academy of Agricultural Sciences, Nanning 530007, China; 2 Department of Biology, Vrije Universiteit Brussel (VUB), 1050 Brussels, Belgium; 3 Institute of Entomology, Guizhou University, Guiyang 550025, China; 4 School of Agricultural Engineering, Guangxi Vocational & Technical College, Nanning 530226, China

**Keywords:** brown planthopper (*Nilaparvata lugens*), volatile organic compounds (VOCs), plant-insect interactions, electroantennography (EAG), Y-tube olfactometer, plant resistance, phytochemical markers

## Abstract

Rice volatiles play a crucial role in mediating resistance to the brown planthopper (*Nilaparvata lugens* Stål, Hemiptera: Delphacidae), a major pest of rice crops. In this study, we analyzed secondary metabolites from rice plants to identify compounds associated with insect behavior. A total of 31 volatile metabolites were detected, among which 16 differed significantly between 51 resistant or susceptible varieties. Fifteen volatiles were more abundant in susceptible plants, while one was enriched in resistant varieties. Electrophysiological (EAG) and Y-tube olfactometer assays revealed that both male and female adults exhibited positive chemotaxis toward five volatiles: Cyclohexanone, 2,2,6-trimethyl-; 3-Cyclohexen-1-one, 3,5,5-trimethyl-; (+)-Isomenthol; Benzoic acid, 2-hydroxy-, methyl ester; and 2-Methoxy-4-vinylphenol. In contrast, male adults were repelled by Benzaldehyde, 3-ethyl-, and 3-Buten-2-one, 4-(2,6,6-trimethyl-1-cyclohexen-1-yl)-. These results indicate that characteristic volatiles serve as functional cues for host selection and may act as phytochemical markers for assessing rice resistance. The findings provide new insights into plant–insect chemical interactions and suggest potential strategies for environmentally friendly pest management, including the use of attractant- or repellent-based approaches and breeding for optimized volatile profiles to control *N. lugens*.

## Simple Summary

The brown planthopper (BPH) is a major pest of rice, causing annual yield losses of over 500,000 tons in China. In the present study, we compared the volatile compounds released by rice varieties that are either susceptible or resistant to BPH. We identified 16 volatile compounds associated with susceptibility or resistance, of which 15 were present at higher levels in susceptible varieties. Further experiments confirmed that five of these compounds attract BPH to susceptible plants. These findings suggest that manipulating attractant or repellent scents, as well as breeding rice varieties with optimized volatile profiles, could be effective strategies for BPH management.

## 1. Introduction

The brown planthopper (*Nilaparvata lugens* Stål, BPH) is one of the most destructive insect pests in rice-producing regions of Asia. BPH feeds on phloem sap, causing reductions in plant height, vigor, productive tillers, and grain filling under light infestations, while heavy infestations can result in “hopperburn”, leading to complete desiccation and plant death. In addition to direct feeding damage, BPH vectors viral diseases, including rice tungro virus, rugged stunt virus, and grassy stunt virus, which can further exacerbate yield losses. In China alone, BPH infestations account for over 500,000 tons of annual rice yield loss [1]. Chemical control of BPH has become increasingly challenging due to the development of insecticide resistance, reducing the efficacy of conventional management strategies. Consequently, cultivating resistant rice varieties has emerged as one of the most effective approaches for sustainable pest management [2,3,4,5]. Previous studies suggest that differences in secondary metabolite composition are a key determinant of varietal resistance to BPH [6,7,8,9]. These metabolites function as chemical signals in the ecosystem and can exert antibiosis effects against insects and pathogens [10,11]. Among secondary metabolites, volatile organic compounds (VOCs) are particularly important. These short-chain hydrocarbons and metabolic byproducts, including aldehydes, ketones, organic acids, esters, and nitrogen- or sulfur-containing compounds, play critical roles in plant–insect interactions [12,13].

During insect–plant coevolution, phytophagous insects have evolved to exploit plant volatiles to locate suitable hosts, making VOCs central to understanding herbivore behavior [14]. In rice, volatiles from the susceptible variety TN1 have been shown to stimulate feeding and growth of BPH and act as attractants, whereas volatiles from the resistant variety IR36 exhibit deterrent effects and can increase BPH mortality [15,16,17,18]. These findings highlight the potential of VOCs as natural modulators of insect behavior and as tools for pest management. The primary biological question of this study is: which specific volatile compounds are responsible for attracting or repelling BPH, and how do these compounds differ between resistant and susceptible rice varieties? We hypothesize that the type and concentration of specific VOCs determine BPH host selection and feeding behavior. To address this, we first characterized and compared the volatile profiles of resistant and susceptible rice varieties. Next, we assessed the electrophysiological sensitivity of male and female BPH to different volatile concentrations using electroantennography (EAG). Finally, we evaluated the behavioral responses of BPH to these compounds using a Y-tube olfactometer assay. By identifying VOCs that modulate BPH behavior, this study provides insights into the chemical mechanisms underlying plant resistance and offers potential strategies for developing environmentally friendly pest management approaches. The results have implications not only for rice protection but also for advancing our understanding of insect–plant chemical ecology, contributing to the broader fields of insect physiology and behavior, secondary plant metabolism, and applied insect–plant sciences.

## 2. Materials and Methods

### 2.1. Rice Plants

Fifty-one rice varieties, including 38 resistant and 13 susceptible lines, obtained from the Plant Protection Research Institute, Guangxi Academy of Agricultural Sciences (GXAAS), China, were used for volatile analysis (Table 1). The resistance of these varieties to *N. lugens* (BPH) was evaluated at the research farm of GXAAS from 2017 to 2020 using a modified standard seed-box screening technique [19,20]. Plant responses were scored on a 0–9 scale: 0 = immune, no visible damage; 1 = highly resistant (HR), partial yellowing of the first leaf; 3 = resistant (R), partial yellowing of the first and second leaves; 5 = moderately resistant (MR), pronounced yellowing or some stunting; 7 = moderately susceptible (MS), mostly wilting but still alive; and 9 = susceptible (S), complete wilting or death of the plant.

### 2.2. Distillation of Rice Plant Volatiles

All rice plants were grown without fertilizer and transplanted into the field at the three-leaf stage. Plants were managed according to standard cultural practices. After 70 days (tillering stage), leaves were collected and transported on ice. Seven replicates were prepared for each variety, with each replicate consisting of seven plants. For volatile extraction, 2 g of leaves per replicate were cut into small pieces and placed into 500-mL distillation flasks. Forty milliliters of water were added to each flask, and the samples were distilled at 40 °C until 25 mL of distillate was obtained. Eight milliliters of n-Hexane were added to the distillate, mixed thoroughly, and the upper organic layer was collected. The extract was dried over anhydrous sodium sulfate, filtered using self-locking filters, and directly analyzed by GC–MS.

### 2.3. GC–MS Conditions

Volatile analysis was performed using a Hewlett-Packard Model 5890 gas chromatograph coupled with a Hewlett-Packard 5970 mass-selective detector. The GC was equipped with a Supelco CV1701 capillary column (30 m × 0.25 mm i.d., 0.25 μm film thickness), and the method followed the previously described procedures with modifications [15]. The oven temperature was programmed as follows: 60 °C (isothermal for 2 min), ramped at 2 °C/min to 120 °C (isothermal for 2 min), then at 4 °C/min to 190 °C, which was held for 20 min. The injector temperature was 250 °C, and 1 μL of sample was injected in split mode (split ratio 10:1). Helium was used as the carrier gas at a flow rate of 1 mL/min. The mass spectrometer operated in electron impact (EI) mode at 70 eV, with the ion source maintained at 220 °C. Mass spectra were recorded over the *m*/*z* range of 45–350, and the GC–MS interface temperature was maintained at 280 °C.

### 2.4. Identification and Quantification of Rice Volatiles

The Wiley Registry of Mass Spectral Data, 7th Edition, was used for data collection and mass spectra processing. Volatile compounds were identified by comparing their mass spectra and retention times with library standards. Each chromatographic peak corresponded to a single volatile, and the peak area was used as an indicator of its relative content.

### 2.5. Electrophysiological Recordings (EAG)

Chemicals: To verify the function of rice volatiles, electroantennography (EAG) was performed to assess antennal responses of *N. lugens* to 11 synthetic compounds. The 11 compounds were: 2-Furan carboxaldehyde, 5-methyl-; 2,4-Heptadienal (E,E)-; Cyclohexanone, 2,2,6-trimethyl-; 3-Cyclohexen-1-one, 3,5,5-trimethyl-; Benzene acetaldehyde; (+)-Isomenthol; Benzaldehyde, 3-ethyl-; Benzoic acid, 2-hydroxy-, methyl ester; 2-Methoxy-4-vinylphenol; 3-Buten-2-one, 4-(2,6,6-trimethyl-1-cyclohexen-1-yl)-; and 2(4H)-Benzofuranone, 5,6,7,7a-tetrahydro. Standards (purity 95–98%) were purchased from Shanghai Yuanye Bio-Technology (Shanghai, China) and dissolved in n-hexane (≥97%; Guangdong Guanghua Sci-Tech, Guangzhou, China). Five concentrations of each compound were tested: 0.01, 0.1, 1, 10, and 100 µg/µL.

Insect rearing: *N. lugens* populations were reared under controlled laboratory conditions (24–26 °C, 60–75% RH) in plastic cages (50 × 50 × 85 cm) containing rice plants covered by nylon mesh. Newly emerged adults (0–3 days old) were collected for bioassays.

EAG setup: Electrodes were prepared from silver wires in two glass capillaries (1.5 mm diameter) filled with saline as reference and recording electrodes. Antennae were excised using fine-tipped forceps, and a small incision was made at the tip. The base and tip were connected to the electrodes following procedures described before [21,22].

Odor stimulation: For each stimulus, 10 μL of chemical solution was applied to a 5 × 0.5 cm filter paper strip and allowed to evaporate for 10 s under a fume hood. The strip was inserted into a 15-cm glass Pasteur pipette to form an odor cartridge. Control pipettes contained 10 μL n-Hexane. The pipette tip was positioned 5 mm from the antennal preparation in a L-shaped glass tube (15 cm × 15 cm diameter). Purified, humidified air (400 mL/min) was pulsed for 0.5 s across the antenna using an IDAC-2 air stimulus controller (Ockenfels Syntech, Buchenbach, Germany). EAG signals were recorded for 10 s, beginning 1 s prior to stimulus onset, with at least 1 min between puffs to allow receptor recovery. Fresh pipettes were prepared daily.

Signal recording and analysis: Analog signals were acquired using a data acquisition controller and analyzed with EAG Pro2.0.2. Maximum deflection after odor stimulation was used as the response measure. For dose-response analyses, EAG amplitudes were normalized relative to the response to 10% n-Hexane to account for sensitivity differences between antennae. Fifteen males and fifteen females were tested, with each concentration sequence repeated six times per individual. All recordings were conducted at room temperature (26 ± 2 °C).

### 2.6. Y-Tube Olfactometer Bioassays

Chemicals and insects: The same 11 synthetic compounds and insects described above were used. Compounds were diluted in dichloromethane, and 20 μL of solution was applied to 5 × 0.5 cm filter papers (Whatman, Maidstone, UK), tested against a control filter paper with 20 μL of n-Hexane.

Y-tube setup: Dual-choice assays were conducted in a horizontally oriented Y-tube olfactometer (15-cm stem, 10-cm side arms, 1.5-cm internal diameter, 60° branching angle). Filter papers with test compounds or controls were placed in each side arm. The Y-tube was placed in a 120 × 50 × 120 cm observation chamber covered by dark cloth. Uniform lighting was provided using a 30-W red lamp (CDLTD-1: 220 V, 50/60 Hz). Purified air was pumped through each arm at 40 mL/min using an electromagnetic air pump (ACO-002, 35 W, Sensen Group, Huaian, China) and a flow meter (LZB-3WB, Xiangjin, Xinghua, China).

Behavioral observations: Individual insects were introduced at the base of the Y-tube and given 5 min to make a choice. A choice was recorded if the insect moved two-thirds along an arm and remained for ≥30 s. Non-responding insects were excluded. After five insects, the Y-tube was cleaned with dichloromethane, and odor sources were switched between arms to avoid positional bias. A total of 60 insects were tested per compound.

Statistical analysis: Data were analyzed using independent-sample *t*-tests for differences between odor treatments. Chi-square (χ^2^) tests were used to compare observed and expected percentages of insects choosing each arm (*p* = 0.05).

## 3. Results

### 3.1. Comparison of Volatile Profiles Between Susceptible and Resistant Rice Plants

Between susceptible and resistant rice varieties (Table 1), a total of 31 volatile compounds were identified (Table 2). Statistical analysis revealed that the concentrations of 16 volatiles differed significantly between the two types. These compounds were: 1-Undecene, 2-methyl-; Furan, 2-ethyl-; 2-Furan carboxaldehyde, 5-methyl-; 2,4-Heptadienal (E,E)-; Cyclohexanone, 2,2,6-trimethyl-; 3-Cyclohexen-1-one, 3,5,5-trimethyl-; Benzene acetaldehyde; (+)-Isomenthol; Benzaldehyde, 3-ethyl-; Benzoic acid, 2-hydroxy-, methyl ester; 1,3-Cyclohexadiene-1-carboxaldehyde, 2,6-; 1-Cyclohexene-1-carboxaldehyde, 2,6,6-tr-; 2-Methoxy-4-vinylphenol; 3-Buten-2-one, 4-(2,6,6-trimethyl-1-cycl; 2(4H)-Benzofuranone,5,6,7,7a-tetrahydro; and CIS,CIS,CIS-7,10,13-Hexadecatrienal. Notably, CIS,CIS,CIS-7,10,13-Hexadecatrienal was present at higher levels in resistant varieties compared with susceptible ones, whereas the remaining 15 compounds were significantly more abundant in susceptible varieties. These differences suggest that specific VOCs may contribute to the observed resistance phenotypes. In particular, volatiles that are more abundant in susceptible varieties could act as feeding or oviposition attractants for *N. lugens*, whereas compounds enriched in resistant plants, such as CIS,CIS,CIS-7,10,13-Hexadecatrienal, may function as repellents or growth inhibitors. This pattern supports our hypothesis that the type and concentration of specific volatiles influence BPH host selection and performance. By linking chemical profiles to insect responses, these results provide a mechanistic basis for the differential susceptibility observed between rice varieties. Understanding these relationships is crucial for the development of pest-resistant cultivars and for designing environmentally friendly pest management strategies that exploit plant-derived chemical cues. The identification of these 16 key volatiles also has broader implications for plant biology. It highlights the role of secondary metabolites in mediating insect–plant interactions, contributing to both ecological signaling and direct defense mechanisms. Furthermore, this work underscores the potential of targeted manipulation of VOC profiles in rice breeding programs aimed at enhancing resistance to herbivores. Future studies exploring the electrophysiological and behavioral responses of BPH to these compounds, as well as their biosynthetic pathways in rice, will further clarify their functional significance.

### 3.2. EAG Response of N. lugens to 11 Compounds

**Figure 1:**
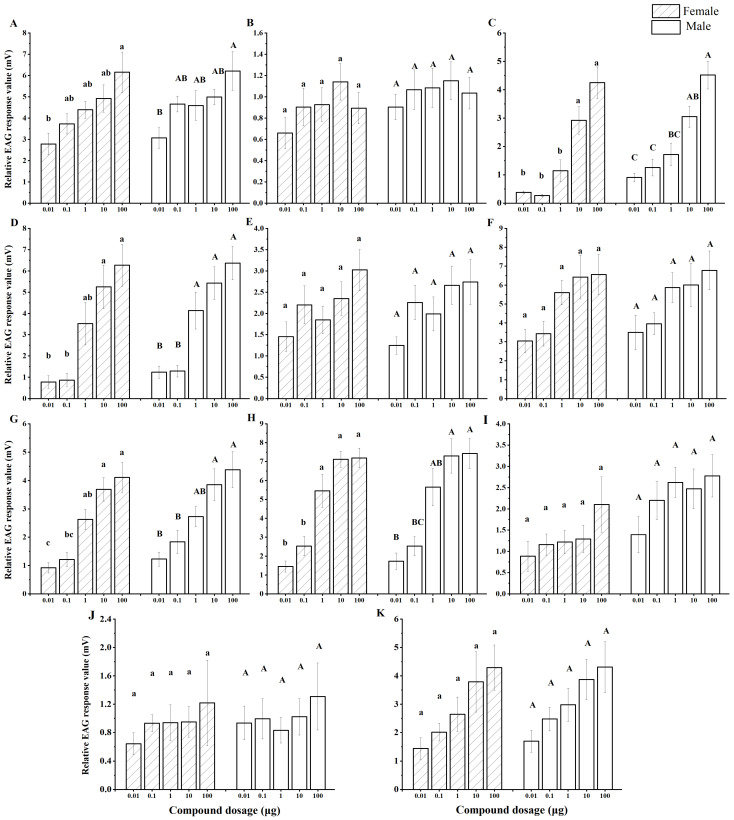
Electroantennography (EAG) responses of *N. lugens* to 11 synthetic rice volatiles at different concentrations. Note: Panels A–K correspond to the following compounds: 2-Furan carboxaldehyde, 5-methyl- (**A**); 2,4-Heptadienal (E,E)- (**B**); Cyclohexanone, 2,2,6-trimethyl- (**C**); 3-Cyclohexen-1-one, 3,5,5-trimethyl- (**D**); Benzene acetaldehyde (**E**); (+)-Isomenthol (**F**); Benzaldehyde, 3-ethyl- (**G**); Benzoic acid, 2-hydroxy-, methyl ester (**H**); 2-Methoxy-4-vinylphenol (**I**); 3-Buten-2-one, 4-(2,6,6-trimethyl-1-cyclohexen-1-yl)- (**J**); and 2(4H)-Benzofuranone, 5,6,7,7a-tetrahydro (**K**). Each point represents the mean ± SE of six antennae. Different lowercase and uppercase letters above the bars indicate significant differences in EAG responses between compounds for female (diagonal bars) and male (hollow bars) adults, respectively (Tukey test, *p* < 0.05).

To investigate the functional role of rice volatiles in resistance to BPH, 11 of the previously identified 16 compounds were synthesized and tested using EAG and Y-tube olfactometer assays. The 11 compounds were: 2-Furan carboxaldehyde, 5-methyl-; 2,4-Heptadienal (E,E)-; Cyclohexanone, 2,2,6-trimethyl-; 3-Cyclohexen-1-one, 3,5,5-trimethyl-; Benzene acetaldehyde; (+)-Isomenthol; Benzaldehyde, 3-ethyl-; Benzoic acid, 2-hydroxy-, methyl ester; 2-Methoxy-4-vinylphenol; 3-Buten-2-one, 4-(2,6,6-trimethyl-1-cyclohexen-1-yl)-; and 2(4H)-Benzofuranone, 5,6,7,7a-tetrahydro. As shown in Figure 1, EAG responses of both female and male *N. lugens* were measured at five concentrations (0.001, 0.1, 1, 10, and 100 μg/μL), and clear concentration-dependent responses were observed, with higher concentrations eliciting stronger antennal signals. A positive correlation was detected between compound concentration and EAG amplitude, indicating that *N. lugens* can perceive these volatiles in a dose-dependent manner. Among the 11 compounds, four, namely 2-Furan carboxaldehyde, 5-methyl-; 3-Cyclohexen-1-one, 3,5,5-trimethyl-; (+)-Isomenthol; and Benzoic acid, 2-hydroxy-, methyl ester, elicited particularly strong responses, with relative voltages exceeding 5 mA. This suggests that these compounds may be especially relevant in mediating insect–host interactions. For the remaining compounds (2,4-Heptadienal (E,E)-; Benzene acetaldehyde; (+)-Isomenthol; 2-Methoxy-4-vinylphenol; 3-Buten-2-one, 4-(2,6,6-trimethyl-1-cyclohexen-1-yl)-; and 2(4H)-Benzofuranone, 5,6,7,7a-tetrahydro), no significant differences in EAG responses were observed between females and males (*p* > 0.05), indicating a similar antennal sensitivity across sexes. Notably, high concentrations of five compounds, namely 2-Furan carboxaldehyde, 5-methyl-; Cyclohexanone, 2,2,6-trimethyl-; 3-Cyclohexen-1-one, 3,5,5-trimethyl-; Benzaldehyde, 3-ethyl-; and Benzoic acid, 2-hydroxy-, methyl ester, elicited significantly stronger EAG responses compared with their low-concentration counterparts. This highlights their potential biological relevance, as these compounds may function as attractants or repellents depending on their abundance in the plant.

Overall, these findings support the hypothesis that specific rice volatiles are differentially detected by *N. lugens* and may underlie the behavioral differences observed between resistant and susceptible varieties. The identification of compounds that trigger strong antennal responses is crucial for understanding the chemical basis of plant resistance and provides candidate molecules for developing environmentally friendly pest management strategies. Further behavioral assays, such as Y-tube olfactometer tests, are needed to confirm whether these electrophysiologically active compounds act as attractants or repellents for BPH.

**Figure 2:**
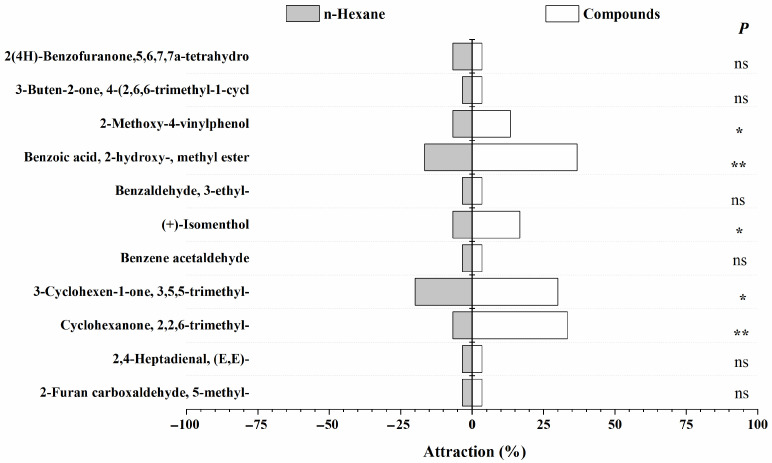
Behavioral responses of female *N. lugens* adults to 11 synthetic rice volatiles in Y-tube olfactometer assays. Note: Significance levels from paired-samples χ^2^ tests are indicated as follows: double asterisk (**), *p* < 0.01, extremely significant; single asterisk (*), *p* < 0.05, significant; ns, *p* > 0.05, not significant. Data are presented as mean ± SE.

**Figure 3:**
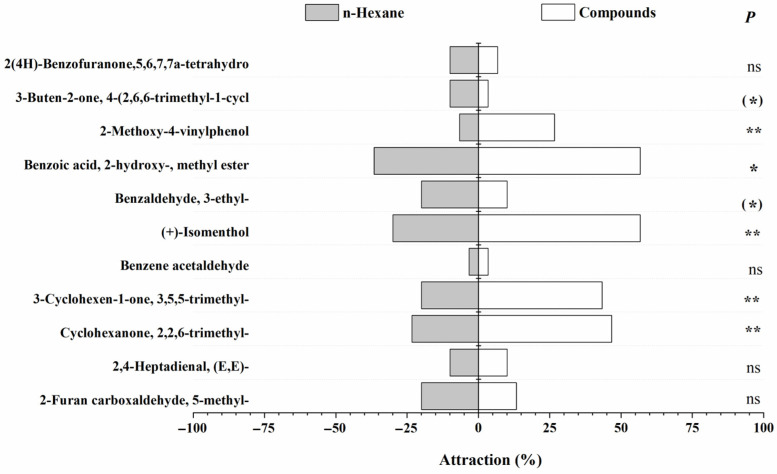
Behavioral responses of male *N. lugens* adults to 11 synthetic rice volatiles in Y-tube olfactometer assays. Note: Significance levels from paired-samples χ^2^ tests are indicated as follows: double asterisk (**), *p* < 0.01, extremely significant; single asterisk (*), *p* < 0.05, significant; ns, *p* > 0.05, not significant. Data are presented as mean ± SE.

Y-tube olfactometer assays revealed that both female and male *N. lugens* adults exhibited positive chemotaxis toward several rice volatiles, including Cyclohexanone, 2,2,6-trimethyl-; 3-Cyclohexen-1-one, 3,5,5-trimethyl-; (+)-Isomenthol; Benzoic acid, 2-hydroxy-, methyl ester; and 2-Methoxy-4-vinylphenol. These compounds elicited clear attraction responses, suggesting their potential role as host-location cues. Interestingly, Benzaldehyde, 3-ethyl-, and 3-Buten-2-one, 4-(2,6,6-trimethyl-1-cyclohexen-1-yl)- displayed sex-specific effects: there was no obvious response to the male adults (Figure 2 and Figure 3). This differential response highlights the nuanced role of VOCs in modulating sex-specific behaviors of *N. lugens*, potentially influencing mating and feeding dynamics. Quantitatively, the strongest attraction for male adults was observed for Cyclohexanone, 2,2,6-trimethyl- (33%, *p* < 0.05), 3-Cyclohexen-1-one, 3,5,5-trimethyl- (30%, *p* < 0.05), and Benzoic acid, 2-hydroxy-, methyl ester (37%, *p* < 0.05). For female adults, Benzoic acid, 2-hydroxy-, methyl ester, and (+)-Isomenthol showed the highest attraction (>50%, *p* < 0.05), followed by Cyclohexanone, 2,2,6-trimethyl- (47%, *p* < 0.05) and 3-Cyclohexen-1-one, 3,5,5-trimethyl- (43%, *p* < 0.05). These results demonstrate that specific volatile compounds mediate host preference and potentially feeding and oviposition behavior in *N. lugens*. Compounds eliciting strong attraction may act as kairomones, guiding the insects to susceptible rice varieties, whereas repellent or sex-specific volatiles could contribute to resistance mechanisms in certain cultivars. Understanding these behavioral responses is critical for developing strategies to exploit plant volatiles in integrated pest management (IPM), such as deploying attractant-baited traps or enhancing the expression of repellent compounds in resistant rice varieties. Overall, the combination of EAG and behavioral assays confirms that both the identity and concentration of specific VOCs are key determinants of *N. lugens* interaction with rice, supporting our hypothesis that volatiles play a central role in mediating insect resistance and susceptibility.

## 4. Discussion

Plant volatile metabolites play critical roles in mediating the host-searching behavior of phytophagous insects [8,23,24]. Rice volatiles are chemically complex, comprising diverse structures and concentrations [25]. For monophagous herbivores, specific volatiles or combinations of compounds can function as a “phytochemical fingerprint,” enabling insects to recognize their host plants [24,26]. Consistent with previous reports, volatiles from the susceptible rice variety TN1 strongly attract BPH [27], and distilled volatile extracts from susceptible varieties also elicit attraction [15].

In the present study, the GC–MS analysis identified 16 volatile compounds associated with rice susceptibility or resistance to BPH. Notably, 15 of these compounds were present at higher levels in susceptible varieties, suggesting that elevated concentrations of specific secondary metabolites may facilitate host localization by BPH. This supports the hypothesis that volatile profiles contribute to varietal susceptibility and highlights the potential for exploiting volatiles in IPM.

EAG and Y-tube olfactometer assays further revealed that five compounds, namely Cyclohexanone, 2,2,6-trimethyl-; 3-Cyclohexen-1-one, 3,5,5-trimethyl-; (+)-Isomenthol; Benzoic acid, 2-Hydroxy-, methyl ester (MeSA); and 2-Methoxy-4-vinylphenol, act as attractants for BPH. These findings provide new insight into the chemical basis of host selection, demonstrating that specific volatiles elicit both electrophysiological and behavioral responses. Previous studies reported that β-Ionone functions as an antifeedant for planthoppers, consistent with the role of inhibitory compounds in our study [25]. Conversely, while Benzaldehyde has been reported to attract *Sogatella furcifera*, our results show a repellent effect on male *N. lugens* adults, highlighting species-specific and possibly stereochemistry-dependent differences in insect response. Such variability underscores the need for precise characterization of volatile structures in ecological studies.

MeSA, a ubiquitous plant metabolite and growth regulator [28,29], is closely related to salicylic acid (SA), a hormone pivotal in plant resistance to pathogens and certain herbivores [30,31,32,33]. MeSA has been reported to exhibit both attractive and repellent activity to BPH [34,35], and can recruit natural enemies following plant injury or infection [36,37,38,39]. Its effect is concentration-dependent [40,41]. In our study, MeSA levels were higher in susceptible varieties, suggesting complex roles in mediating insect–host interactions. Further studies are required to dissect the specific functions and interactions of MeSA and other volatiles in rice defense.

We also want to note that here some inconsistencies were observed between GC–MS quantification and behavioral responses. For instance, Benzaldehyde, 3-Ethyl-, and 3-Buten-2-one, 4-(2,6,6-trimethyl-1-cyclohexen-1-yl)- repelled male adults in Y-tube assays despite being present at higher concentrations in susceptible varieties. Such discrepancies may result from synergistic or antagonistic interactions among compounds in the plant matrix, which cannot be fully captured by single-compound assays. Elucidating these interactions represents an important area for future research.

Several of the identified volatiles, including 3-Cyclohexen-1-one, 3,5,5-Trimethyl-; MeSA; and 2-Methoxy-4-vinylphenol, are precursors or intermediates for the synthesis of pesticides and other bioactive compounds. Benzaldehyde, 3-ethyl-, serves as an intermediate for herbicides and plant growth regulators, while 3-Buten-2-one, 4-(2,6,6-trimethyl-1-cyclohexen-1-yl)- is used in perfumery and vitamin A synthesis. Understanding the mode of action of these volatiles on BPH could inform the development of novel pest control agents.

Volatiles from resistant rice varieties have been reported to alter reproductive behavior and increase mortality of *S. furcifera* [42], and they serve as key cues enabling insects to locate host plants [43,44,45]. Differences in plant resistance are often reflected in distinctive secondary metabolite profiles, suggesting that characteristic volatiles could serve as markers or “phytochemical fingerprints” for resistance assessment. Integrating volatile profiling with marker-assisted selection may accelerate breeding of resistant varieties. Despite these advances, significant gaps remain. The precise mechanisms by which individual or combinations of volatiles influence BPH behavior are not fully understood. The effects of concentration, synergistic interactions, and stereochemistry require systematic investigation. Moreover, the ecological relevance of laboratory findings needs validation under field conditions. Future studies should focus on the functional characterization of these compounds, their biosynthetic pathways, and their integration into sustainable pest management strategies. Overall, this study provides novel insights into the role of rice volatiles in BPH resistance and highlights avenues for both basic research and applied agricultural innovation.

## 5. Conclusions

In the present study, 16 volatile compounds from rice were associated with susceptibility or resistance to *N. lugens*. Among these, fifteen volatiles were present at higher levels in susceptible varieties. EAG and behavioral (Y-tube olfactometer) assays further demonstrated that five of these compounds act as attractants for BPH. These findings provide new insights into the chemical cues underlying host selection by BPH and highlight specific rice volatiles as key mediators of insect–plant interactions. Understanding the functional roles and mechanisms of these characteristic compounds could inform the development of environmentally friendly, targeted pest management strategies, including the use of attractant-based traps or breeding of varieties with optimized volatile profiles to reduce pest pressure.

## Figures and Tables

**Table 1:** List of 51 rice varieties selected for GC–MS analysis of volatile metabolites.

No.	Variety	Scale of Resistance	No.	Variety	Scale of Resistance	No.	Variety	Scale of Resistance
1	08BPH451-1	1	18	08BPH491-1	18	35	08BPH639-1	1
2	08BPH451-2	3	19	08BPH493-1	19	36	08BPH 640	3
3	08BPH453-1	1	20	08BPH565-1	20	37	2183	3
4	08BPH457-1	5	21	08BPH566-1	21	38	BPHR54	1
5	08BPH457-2	5	22	08BPH 69-5	22	39	08GAN-1	7
6	08BPH459-1	5	23	08BPH570-2	23	40	08GAN-2	7
7	08BPH463-1	3	24	08BPH571-1	5	41	08GAN-3	7
8	08BPH463-2	3	25	08BPH574-1	3	42	08GAN-4	7
9	08BPH465-1	1	26	08BPH575-1	1	43	08GAN-5	7
10	08BPH465-2	1	27	08BPH582-1	1	44	08GAN-6	7
11	08BPH466-1	3	28	08BPH584-1	5	45	08GAN-7	7
12	08BPH471-1	1	29	08BPH602-1	3	46	08GAN-8	7
13	08BPH471-2	3	30	08BPH603-1	5	47	08GAN-9	7
14	08BPH479-1	5	31	08BPH609-1	5	48	08GAN-10	7
15	08BPH480-1	5	32	08BPH630-1	3	49	08GAN-11	7
16	08BPH488-1	1	33	08BPH 30-2	3	50	08GAN-12	7
17	08BPH489-1	5	34	08BPH631-1	1	51	TN1	9

**Table 2:** Comparison of volatile compound contents between resistant and susceptible rice varieties.

No.	Retention Time (min)	Volatiles Name	Mean Volatiles Content of Resistant Plants	Mean Volatiles Content of Susceptible Plants	Ratio Between Resistant and Susceptible Plants
1	4.39	1-Undecene, 2-methyl-	135,471 ± 13,087	229,647 ± 33,145 **	0.590
2	4.78	Oxime-, methoxy-phenyl-	138,901 ± 7276	163,230 ± 13,218	0.851
3	5.24	Furan, 2-ethyl-	221,178 ± 30,310	467,724 ± 75,007 **	0.473
4-1	6.33	2-Furan carboxaldehyde, 5-methyl-	220,807 ± 23,233	353,092 ± 46,491 **	0.625
5	6.38	Benzaldehyde	171,992 ± 14,065	187,475 ± 23,700	0.917
6-2	7.16	2,4-Heptadienal, (E,E)-	124,921 ± 14,077	218,112 ± 11,651 **	0.573
7-3	8.11	Cyclohexanone, 2,2,6-trimethyl-	65,499 ± 5277	87,576 ± 5714 *	0.748
8-4	8.24	3-Cyclohexen-1-one, 3,5,5-trimethyl-	93,062 ± 6415	170,519 ± 28,143 **	0.546
9-5	8.29	Benzene acetaldehyde	678,098 ± 33,291	874,977 ± 61,462 **	0.775
10	9.29	Phenol, 2-methoxy-	218,904 ± 49,374	187,568 ± 35,332	1.167
11-6	9.92	(+)-Isomenthol	145,940 ± 10,679	240,877 ± 29,589 **	0.606
12	10.74	2,6,6-Trimethyl-2-cyclohexene-1,4-dione	64,298 ± 6399	67,301 ± 9047	0.955
13-7	11.20	Benzaldehyde, 3-ethyl-	109,405 ± 10,434	178,486 ± 24,249 **	0.613
14	11.50	Benzaldehyde, 2,4-dimethyl-	101,193 ± 5717	128,723 ± 34,856	0.786
15	11.76	Naphthalene	273,545 ± 27,294	287,657 ± 23,976	0.951
16-8	11.91	Benzoic acid, 2-hydroxy-, methyl ester	86,973 ± 10,872	137,209 ± 22,907 *	0.634
17	12.09	1,3-Cyclohexadiene-1-carboxaldehyde, 2,6	82,285 ± 4404	136,286 ± 20,571 *	0.604
18	12.42	Benzofuran, 2,3-dihydro-	181,014 ± 10,586	233,134 ± 29,543	0.776
19	12.59	1-cyclohexene-1-carboxaldehyde, 2,6,6-tr	190,926 ± 11,367	325,316 ± 27,521 **	0.587
20	12.77	Benzothiazole	213,053 ± 16,162	208,055 ± 21,535	1.024
21	13.59	1-Cyclohexene-1-acetaldehyde,2,6,6-trim	69,259 ± 11,297	81,177 ± 8268	0.853
22	14.11	Phenol, 4-ethyl-2-methoxy-	130,794 ± 9392	153,840 ± 16,660	0.850
23	14.68	Indole	136,367 ± 25,300	127,800 ± 13,472	1.067
24-9	15.24	2-Methoxy-4-vinylphenol	2,021,727 ± 111,544	3,222,740 ± 283,773 **	0.627
25	18.16	2-Buten-1-one,1-(2,6,6-trimethyl-1-cycl	55,675 ± 9395	69,517 ± 7248	0.801
26-10	20.08	3-Buten-2-one, 4-(2,6,6-trimethyl-1-cycl	444,255 ± 46,599	909,326 ± 108,235 **	0.489
27	20.73	Butylated hydroxytoluene	486,276 ± 49,118	528,301 ± 36,068	0.920
28-11	21.37	2(4H)-Benzofuranone,5,6,7,7a-tetrahydro	157,046 ± 10,648	266,567 ± 15,642 **	0.589
29	22.59	Megastigmatrienone	221,997 ± 22,778	249,617 ± 22,175	0.889
30	28.96	1,2-Benzene dicarboxylic acid, bis(2-meth	113,750 ± 11,191	91,694 ± 8212	1.241
31	29.83	CIS,CIS,CIS-7,10,13-Hexadecatrienal	244,550 ± 26,429 **	139,635 ± 17,718	1.751

Note: The asterisks (*) in the column data (peak areas from chromatograms, mean ± SE) indicate significant differences at *p* = 0.05, and double asterisks (**) indicate significant differences at *p* = 0.01, based on a *t*-test.

## Data Availability

The original contributions presented in this study are included in the article. Further inquiries can be directed to the corresponding authors.
